# A multilingual heterogeneous knowledge graph framework for curriculum–vacancy–MOOC alignment: edge-weight regression with heterogeneous graph transformers for educational recommendation

**DOI:** 10.3389/frai.2026.1906883

**Published:** 2026-10-15

**Authors:** Sandugash Serikbayeva, Madina Sambetbayeva, Valiya Ramazanova, Zhanar Lamasheva, Aigerim Yerimbetova, Valentina Makhatova, Zhanna Sadirmekova

**Affiliations:** 1 Department of Information Systems, L.N. Gumilyov Eurasian National University, Astana, Kazakhstan; 2 M. Utemisov West Kazakhstan University, Center of Information Technologies, Oral, Kazakhstan; 3 Institute of Information and Computational Technologies CS MSHE, RK, Almaty, Kazakhstan; 4 School of Engineering and Information Technology, META University, Almaty, Kazakhstan; 5 Atyrau University named after Kh. Dosmukhamedov, Atyrau, Kazakhstan; 6 School of Software Engineering, Astana IT University, Astana, Kazakhstan

**Keywords:** curriculum–job alignment, educational data mining, educational recommendation, heterogeneous graph neural networks, heterogeneous graph transformer, knowledge graph, link-weight regression, MOOC

## Abstract

**Introduction:**

Digitalisation and artificial intelligence have widened the gap between competencies certified by university curricula and skills demanded by employers. MOOCs could close this gap, but existing recommenders rarely connect what a programme teaches, what the market requires, and what online courses develop within a single semantic structure.

**Methods:**

We built a multilingual heterogeneous knowledge graph (KG) that integrates university programmes, IT vacancies from hh.kz, and Coursera MOOCs in English, Russian, and Kazakh. A property-graph ontology was formalised and instantiated automatically. Skills were aligned with a fine-tuned multilingual Sentence Transformer (paraphrase-multilingual-mpnet-base-v2), and agglomerative clustering formed skill communities. Recommendation was cast as edge-weight regression over a competency-to-course relation and solved with GraphSAGE, HAN, and HGT encoders.

**Results:**

On a held-out 20% test graph, HGT achieved the best ranking quality (NDCG@10 = 0.714; with short meta-paths NDCG@10 = 0.987, RMSE = 0.108), outperforming GraphSAGE and HAN. Applied to the “Information Systems” programme of a Kazakhstani university, the system expanded the market-aligned competency profile by +36.2% with five top-ranked courses and by +170% with the full catalogue.

**Discussion:**

Edge-weight regression on a cross-domain heterogeneous KG produces front-loaded, actionable course recommendations for curriculum alignment. All datasets, models, graph snapshots, and code are released for reproducibility.

## 1 Introduction

The half-life of professional skills is shrinking. The World Economic Forum estimates that a large share of the global workforce will require reskilling within a few years as technological skill profiles are transformed, and analysts repeatedly argue that higher education must build programmes around the competencies that employers actually demand rather than around slowly revised standards (World Economic Forum, 2023; McKinsey & Company, 2023). In Kazakhstan, the same tension is documented in national labour-market reporting: University programmes are updated slowly and do not always reflect current demand (Ministry of Labor and Social Protection of the Population of the Republic of Kazakhstan, 2024). The consequence is an educational skills mismatch—a structural divergence between the competencies a graduate is certified to possess and the skills that employers list in live vacancies.

The problem is exacerbated by the current technological moment. As artificial intelligence becomes embedded across the economy, national policy in Kazakhstan explicitly frames digital transformation and human-capital development as intertwined priorities and recognises that AI can analyse and recommend promising development paths. Within a university, this calls for a new paradigm of information support for the educational process: one that does not merely digitise administration but actively intellectualises the institution’s information resources so that internal artefacts (programmes, syllabi, competency maps) and external ones (vacancies, MOOCs, skill classifiers) are connected rather than siloed. Today, these resources are typically linked only through manual organisational effort; we argue that they should instead be linked semantically, using NLP to establish meaning-preserving correspondences and knowledge graphs to structure the exchange.

Massive open online courses (MOOCs) are an obvious instrument for closing this gap. Platforms such as Coursera offer thousands of courses, and Kazakhstani universities have begun to embed MOOC modules into credit-bearing courses under integrated assessment models. Yet the abundance of courses is itself a problem: with thousands of overlapping offerings, learners and curriculum designers cannot easily identify which courses develop the specific competencies that are simultaneously (i) missing from a programme and (ii) demanded by the market. Educational recommendation is therefore not a generic item-ranking task; it is a cross-domain alignment task that must reason jointly over three heterogeneous information sources.

Knowledge graphs (KGs) are a natural substrate for such reasoning because they represent entities and typed relations from different domains in a single structure and expose latent connections between them. When combined with natural language processing (NLP) for semantic alignment and with graph neural networks (GNNs) for inference, KGs can power recommenders that exploit domain knowledge rather than interaction histories, which is essential in cold-start educational settings where per-user click data are unavailable (Shiwen et al., 2023; Fayyaz et al., 2020; Chicaiza and Valdiviezo-Diaz, 2021). However, three limitations recur in the literature and motivate this work.

### 1.1 Research gap

First, most prior systems integrate only one or two domains, typically vacancies and skills or courses and skills, whereas the alignment problem fundamentally couples three domains: curricula, vacancies, and MOOCs. Second, the integration of skills across sources is almost always monolingual (predominantly English) and relies on monolingual embedding models, which is inadequate for trilingual educational ecosystems such as Kazakhstan’s (English, Russian, Kazakh). Third, GNN-based recommenders in education overwhelmingly use classical homogeneous architectures (GCN, GAT, GraphSAGE) and frame the task as binary link prediction; the use of heterogeneous graph transformers, and in particular the formulation of recommendation as continuous edge-weight regression over a cross-domain heterogeneous educational KG, is essentially unexplored.

This study addresses that gap with a single, reproducible framework. We design and formalise a property-graph ontology spanning the three domains; we align competencies, requirements, and skills using a multilingual Sentence Transformer that we fine-tune on a purpose-built trilingual paraphrase corpus; we densify the graph with skill communities discovered by agglomerative clustering; and we recast educational recommendation as the regression of a continuous relevance score on a learner-competency-to-course edge, which we solve with GraphSAGE, HAN, and HGT encoders. We then deploy the best model in a working prototype and quantify, on a real programme, how much it expands the market-aligned competency profile.

### 1.2 Research questions

The study is organised around five research questions (RQs):

*RQ1*: Can a single property-graph ontology coherently integrate university curricula, labour-market vacancies, and MOOCs into one heterogeneous, multilingual knowledge graph?

*RQ2*: Which embedding model best captures the semantic equivalence of short, noisy skill phrases across English, Russian, and Kazakh, and does domain fine-tuning of a multilingual Sentence Transformer improve cross-lingual alignment?

*RQ3*: Which clustering algorithm yields the highest-quality skill communities for graph densification, and does embedding fine-tuning improve cluster quality?

*RQ4*: When educational recommendation is posed as edge-weight regression on a cross-domain heterogeneous KG, how do GraphSAGE, HAN, and HGT encoders compare on regression and ranking metrics, and how does meta-path length affect generalisation?

*RQ5*: Does the resulting system measurably increase the market-aligned completeness of a real educational programme, and how many courses are needed to capture most of that gain?

### 1.3 Contributions

In answering these questions, the study makes the following contributions:

C1. A formal property-graph ontology and an automated construction pipeline for a tri-domain heterogeneous educational KG that unifies curricula, vacancies, and MOOCs; to our knowledge, this is the first educational KG to semantically couple all three domains in a multilingual setting (English, Russian, Kazakh).

C2. A multilingual semantic skill-integration method built on a Sentence Transformer fine-tuned with a multiple-negatives-ranking objective on a new trilingual paraphrase corpus, together with a released English skill-pair evaluation set; fine-tuning improves Russian and Kazakh alignment without degrading English.

C3. A skill-community densification step based on a controlled comparison of four clustering algorithms, in which agglomerative hierarchical clustering is selected and shown to improve after embedding fine-tuning (silhouette 0.271 → 0.285; expert-judged erroneous clusters 19.7% → 14.0%).

C4. The first formulation of educational recommendation as continuous edge-weight regression on a cross-domain heterogeneous KG, with a controlled comparison of GraphSAGE, HAN, and HGT encoders under encoder–decoder architectures; the HGT encoder is the strongest ranker, and a meta-path-length ablation explains its behaviour.

C5. An open, reproducible Flask/Neo4j prototype and a programme-level evaluation showing a measurable expansion of a real curriculum’s market-aligned competency profile (+36.2% from five courses; +170% in full), with a full release of data, models, graph snapshots, and code.

The remainder of the article is structured as follows. Section 2 reviews related work. Section 3 describes the materials and datasets. Section 4 presents the proposed framework: KG construction, semantic integration, skill-community detection, and the recommendation model. Section 5 details the experimental setup; Section 6 reports results, including statistical comparisons and ablations. Section 7 describes the prototype and the reproducibility protocol. Sections 8–9 discuss implications, limitations, and conclusions. All in-text references to tables, figures, equations, and sections throughout the manuscript are cross-referenced to their labelled objects (Equations 1–17, Tables 1–11, Figure 1, and Sections 1–10) so that the numbering updates consistently with any future revisions.

**Table 1:** Node counts in the heterogeneous KG by class.

Node class	Description	Train	Test
CUA	User competency descriptions	125	30
SkillMastered	Mastered vacancy skills	371	
Vacancy	Labour-market vacancy titles	3,478	
Skill	Unmastered vacancy skills	2,676	
MSkill	Course skill names	791	
MCourse	MOOC course names	877	

The Test column is given only where a separate test set exists.

**Table 2:** Edge types in the heterogeneous KG (direct edges; reverse edges added for message passing).

Relation	Direction	Train	Test
IS_SIMILAR_TO	CUA → SkillMastered	155	37
GROUPED_WITH_SKILL	SkillMastered–SkillMastered	778	
REQUIRE_SKILL	SkillMastered ← Vacancy	7,600	
REQUIRE_SKILL	Vacancy → Skill	17,749	
IS_SIMILAR_TO2	Skill → MSkill	299	
GROUPED_WITH_MSKILL	MSkill–MSkill	2,622	
DEVELOPED_BY	MSkill → MCourse	6,074	
metapath_0	CUA → … → MCourse (with both communities)	12,274	1,886
metapath_1	CUA → … → MCourse (skill community)	7,659	1,267
metapath_2	CUA → … → MCourse (mskill community)	17,856	2,710
metapath_3	CUA → … → MCourse (no community)	10,195	1,615
CONNECTED_TO	CUA → MCourse (training labels)	24,108	4,875

The Test column is given only where a separate test set exists.

**Table 3:** Embedding-model comparison on the in-house skill-pair set and the public STSB set.

Model	P (skills)	R (skills)	F1 (skills)	P (STSB)	R (STSB)	F1 (STSB)
BERT	0.548	0.965	0.699	0.553	1.000	0.712
Word2Vec	0.991	0.634	0.773	0.580	0.988	0.731
paraphrase-multilingual-mpnet-base-v2	0.983	0.816	0.892	0.667	0.991	0.797

**Table 4:** Selected pre−/post-fine-tuning correlations on public benchmarks (higher is better).

Measure/language	EN before	EN after	RU before	RU after
Cosine–Pearson	0.854	0.855	0.813	0.822
Cosine–Spearman	0.868	0.860	0.825	0.824
Dot product–Pearson	0.827	0.823	0.717	0.757
Dot product–Spearman	0.823	0.811	0.696	0.736
Euclidean–Pearson	0.865	0.861	0.808	0.821

**Table 5:** Clustering quality by silhouette coefficient (out-of-the-box embeddings).

Algorithm	Best key parameter	Best silhouette
Agglomerative (average, cosine)	threshold = 0.29	0.2713
K-Means	*k* = 1,400	0.2429
DBSCAN	ε = 0.55	0.2092
HDBSCAN		0.0739

**Table 6:** Agglomerative clustering before and after embedding fine-tuning (3,047 skills).

**Fine-tuning**	**Threshold**	**Clusters**	**Mean silhouette**	**Singletons**	**Erroneous**
Before	0.29	1,121	0.2713	19.2%	19.7%
After	0.37	1,407	0.2851	24.0%	14.0%

**Table 7:** Test-set quality of the three encoders (link-weight regression).

Model	*k*	Precision	Recall	MAP	NDCG
GraphSAGE (RMSE 0.221)	5	0.307	0.217	0.296	0.540
GraphSAGE	10	0.293	0.302	0.308	0.590
GraphSAGE	20	0.282	0.477	0.336	0.650
HAN (RMSE 0.204)	5	0.400	0.233	0.387	0.581
HAN	10	0.311	0.307	0.345	0.614
HAN	20	0.266	0.420	0.340	0.652
HGT (RMSE 0.259)	5	0.557	0.306	0.574	0.697
HGT	10	0.486	0.427	0.551	0.714
HGT	20	0.384	0.592	0.523	0.739

**Table 8:** HGT recommender by meta-path length (test set).

Meta-path group	*k*	Precision	Recall	MAP	NDCG
Short: CUA–MasteredSkill–Vacancy	5	0.793	0.578	0.967	0.980
Short	10	0.647	0.734	0.967	0.987
Intermediate: Vacancy–Skill–MSkill–MCourse	5	0.728	0.605	0.921	0.959
Intermediate	10	0.588	0.755	0.918	0.964
Long: metapath_0–3 (composite)	5	0.550	0.305	0.570	0.716
Long	10	0.486	0.423	0.558	0.741

**Table 9:** Positioning against representative link-weight prediction approaches.

Approach	Graph/data	Metric	Value
Fully-connected NN/Model R (Reimers and Gurevych, 2019)	Homogeneous weighted	MSE	0.02
Line-graph LWP (Reimers and Gurevych, 2020)	Homogeneous weighted	RMSE	0.06–0.18
GraphRec (Liang et al., 2025)	Heterog. weighted (social)	RMSE	0.24–0.26
HinSAGE (Hou and Holder, 2018)	Heterog. weighted (IMDB)	RMSE	0.25
SBERT + GraphSAGE (Liu et al., 2024)	Heterog. weighted (IMDB)	RMSE	0.22
This work (SBERT + HGT)	Heterog. semi-weighted, 3-domain, multilingual	RMSE/MSE	0.1–0.2/0.02–0.04

**Figure 1:**
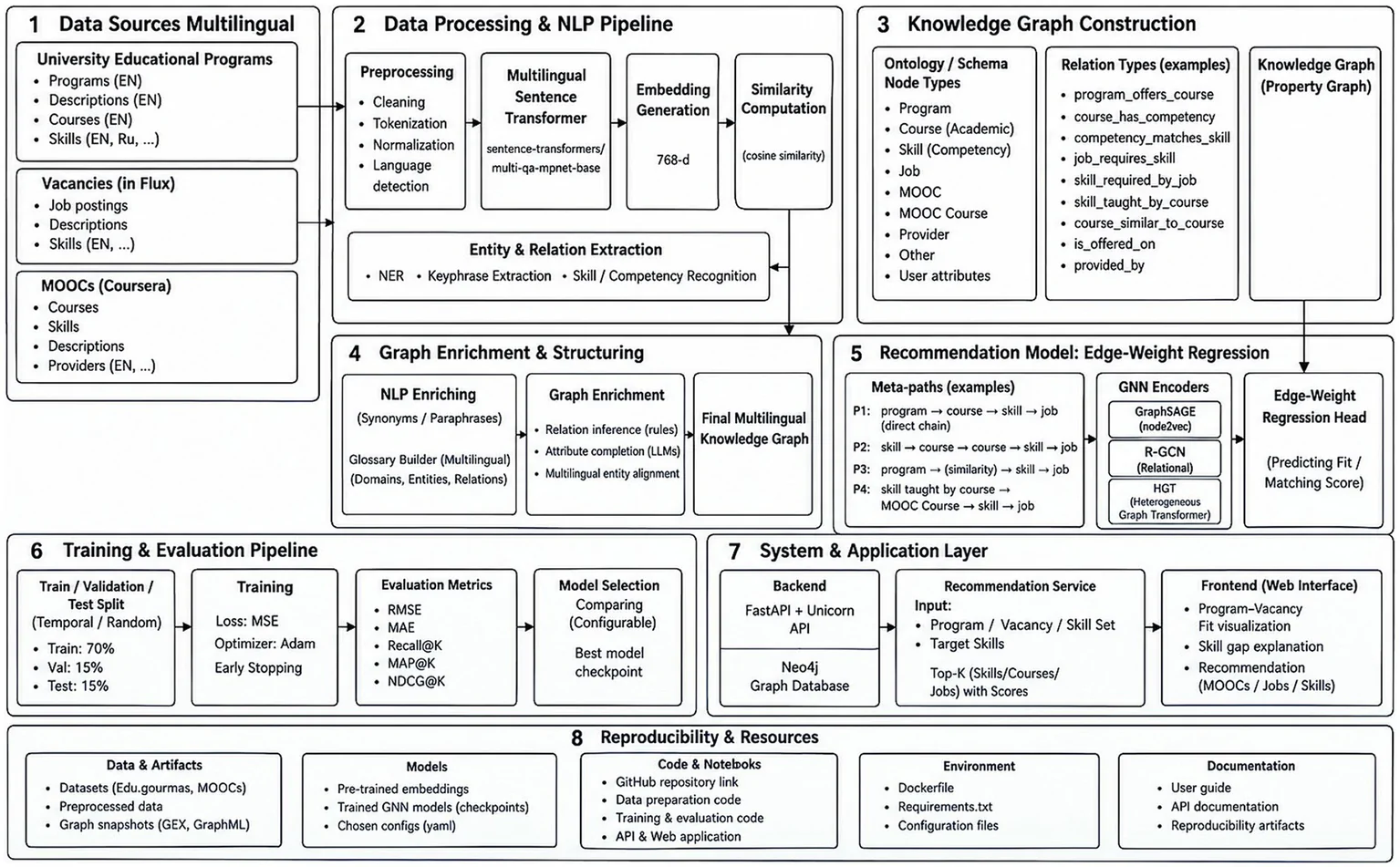
Architecture of the multilingual heterogeneous knowledge graph framework for Curriculum–Vacancy–MOOC alignment and educational recommendation.

## 2 Related work

### 2.1 Knowledge graphs in education

Knowledge graphs have evolved from a symbolic, ontology-centred technology to hybrid symbolic–vector representations that combine logical semantics, data-model structure, and machine-learning embeddings (Dong et al., 2022; Ramazanova et al., 2024; Hogan et al., 2021). In education, they have been applied to interactive learning-resource exploration (Shiwen et al., 2023), remote-learning support with language models (Chaudhri et al., 2022), scientific-resource management (Nair et al., 2022), personalised learning paths (Chi et al., 2018; Rizun, 2019), and domain learning environments in subjects ranging from nuclear engineering (Sun et al., 2021) to mathematics (Telnov and Korovin, 2019; Zhao et al., 2019), cybersecurity (Chen et al., 2018), computer science (Agrawal et al., 2022), and databases (Su and Zhang, n.d.). A recurring classification, organised by stakeholder (students, instructors, administrators) and by purpose, shows that most systems serve as domain-learning environments, content-structuring aids, or learning-path builders (Qin et al., 2020). The dominant limitation is scope: the vast majority of these systems operate within a single narrow domain and do not attempt cross-domain integration with the labour market.

### 2.2 Curriculum–job alignment and skill integration

A second strand integrates skills across sources using language models and, partially, knowledge graphs. Representative work draws on vacancies, résumés, occupational classifiers (ESCO, O*NET/DWA), university courses, and MOOCs, typically combining two domains at a time (Ramazanova et al., 2024; Giabelli et al., 2021; Fettach et al., 2022; Weichselbraun et al., 2022; Zhu et al., 2022; Börner et al., 2018). Early systems used FastText or Word2Vec; later ones transitioned to BERT, Sentence-BERT, and large language models. Two features are nearly universal and motivate our design choices: the corpora and embedding models are monolingual (overwhelmingly English), and when a recommendation mechanism is present, it is usually a graph query, cosine search, or a shallow neural model rather than a heterogeneous GNN. Comparative analysis (Section 6) confirms that systems integrating three domains in a multilingual setting with a heterogeneous-GNN recommender are absent from this literature.

### 2.3 MOOC recommendation

Recommender systems are traditionally divided into content-based filtering, which matches items to a user/item profile; collaborative filtering, which exploits behaviour and ratings but suffers from cold start; and hybrids. The field has since broadened to include demographic, context-aware, utility-based, and knowledge-based filtering (Chau, 2024; Frej et al., 2024; Fayyaz et al., 2020). Knowledge-based and KG-driven approaches are especially attractive in education because they recommend based on domain knowledge rather than rating histories, mitigating cold start and sparsity while improving the diversity of recommended items. Within KG-based recommendation, four sub-families are commonly distinguished: Ontology-based, linked-open-data-based, embedding-based, and path-based, and the preference function may be realised by support-vector methods, factorisation machines, deep neural networks, NLP-based neural models, reinforcement learning, or graph neural networks (Frej et al., 2024; Fayyaz et al., 2020). Graph-based MOOC recommenders increasingly use GNNs and contrastive learning, but they typically treat courses and learners (or courses and concepts) within a single domain and predict the existence of a link rather than graded relevance. Our framework differs by grounding MOOC recommendation in a tri-domain graph and by predicting a continuous relevance weight that encodes how strongly a course closes a market-relevant competency gap.

### 2.4 Heterogeneous graph neural networks

For recommendation, KG vectorisation is commonly organised into GNN-based methods, multi-view graphs, and multi-layer graphs (Chicaiza and Valdiviezo-Diaz, 2021; Dong et al., 2022). GNN-based models (e.g., KGLN, KHGCN, and FEKGNN) enable fast parallel computation and capture graph dynamics but can undervalue popular items under negative sampling; multi-view models preserve relational multiplicity at the cost of node-importance weighting; and multi-layer models support cross-domain recommendation but require expert layer modelling. Within heterogeneous GNNs, GraphSAGE (Sun et al., 2021) provides an inductive baseline that aggregates neighbourhood features; HAN (Yousefzadeh et al., 2025) introduces node-level and semantic-level (meta-path) attention; and HGT (Hamilton et al., 2017) parameterises attention and message passing by node and edge types, scaling to large heterogeneous graphs without hand-crafted meta-paths. These architectures are well established for node classification and binary link prediction, but their use for continuous edge-weight regression in a cross-domain educational KG has not, to our knowledge, been reported.

Three limitations of the existing heterogeneous-GNN literature are particularly relevant to the educational setting addressed here. First, the dominant evaluation paradigm is binary or implicit link prediction, in which a candidate edge is scored as present or absent; this framing is poorly matched to educational recommendation, where the pedagogically meaningful quantity is the degree to which a course covers the competencies a learner lacks—a graded rather than binary relationship. Second, meta-path-based models such as HAN depend on a fixed catalogue of hand-specified meta-paths, which both inject domain assumptions into the model and inflate the number of edge types that must be materialised; in a tri-domain graph, the combinatorial growth of plausible meta-paths makes exhaustive enumeration impractical and selective enumeration arbitrary. Third, multi-view and multi-layer formulations, while expressive, presuppose that the layers or views are known and cleanly separable—an assumption that breaks down when the same skill concept is expressed heterogeneously across institutional curricula, employer postings, and course catalogues in three languages. The framework proposed in this study responds to these three limitations directly: it casts recommendation as continuous edge-weight regression, it uses a type-parameterised attention mechanism (HGT) that learns from node and edge types without requiring a curated meta-path set, and it resolves cross-domain lexical heterogeneity upstream through a fine-tuned multilingual encoder before the graph is presented to the GNN.

### 2.5 Positioning of this work

Against this background, the present study is distinguished by three features taken together: (i) breadth of integration across three domains (curricula, vacancies, MOOCs) rather than one or two; (ii) multilingualism, with English, Russian, and Kazakh handled by a fine-tuned multilingual Sentence Transformer; and (iii) recommendation architecture, employing a heterogeneous GNN (HGT) applied to edge-weight regression. The comparative tables in Section 6 make this positioning explicit relative to both the link-weight-prediction literature and the curriculum–job integration literature.

## 3 Materials and datasets

The framework consumes three complementary corpora, deliberately chosen so that each captures a distinct facet of the alignment problem and allows for cross-referencing at the level of skills. Educational programmes encode the official competencies and learning outcomes that define a graduate’s certified profile. Live vacancies provide a continuously updated picture of demanded skills, in contrast to occupational standards and classifiers, which lag behind the market. MOOCs constitute the supplementary-learning segment that can close identified gaps. Jointly, the three corpora make it possible to detect which competencies a programme lacks relative to current demand and which courses develop exactly those competencies; no single corpus supports this inference alone.

### 3.1 University educational programmes

The first (internal) corpus consists of descriptive texts of university educational programmes (curricula) in .docx format. These semi-structured documents contain, in Kazakh, Russian, and/or English, the programme code and title, the competency map, intended learning outcomes, and discipline annotations. After the manual removal of non-essential text, sentences are split at the delimiters “,” “;” and “.” into phrases, which are then cleaned and normalised. Each phrase becomes a node describing a competency, learning outcome, or annotation (the CUA class), attached to its programme. Granularity varies by source field: discipline annotations range from a discipline or module title down to individual topic-level phrases; the competency map distinguishes between general (soft, cross-cutting) competencies and professional (hard, subject-specific) competencies aligned to the state educational standard; and learning-outcome phrases are further separated by cognitive level (knowledge, skill, and applied-skill/competency statements, in the spirit of a Bloom-type taxonomy) and by their source discipline. Across the programmes processed for this study, this yields 125 distinct CUA phrases spanning these categories (Table 1), each retaining a back-reference to its originating discipline and programme, so that the granularity of the source annotation is preserved in the graph rather than collapsed at ingestion.

### 3.2 Labour-market vacancies

The second (external) corpus comprises IT and adjacent vacancies retrieved from the HeadHunter API (https://api.hh.ru/vacancies). Each vacancy is a structured JSON object exposing the title, profession, region, employer, experience, salary, publication date, the free-text requirement and responsibility fields, and a key_skills list. Vacancies were collected for 25 IT professions (analyst, developer, DevOps engineer, data scientist, BI/data analyst, QA engineer, system administrator, information-security specialist, product/project manager, and others) across 177 localities in Kazakhstan over February–March 2024. The skill-mining corpus contains 5,248 vacancies and 3,047 unique skills; a separate programme-level test corpus (Section 6.6) contains 1,184 vacancies with 8,940 unique skills.

### 3.3 MOOCs

The third (external) corpus consists of Coursera course descriptions in English, Russian, and Kazakh in the Computer Science, Data Science, and Information Technology topics, scraped using BeautifulSoup. Course cards provide the title, provider, programme type, language, rating, level, and duration; course pages additionally expose acquired skills, module titles and descriptions, and lesson items. These map onto the MCourse, MSkill, MModule, and MLesson classes.

### 3.4 Evaluation and fine-tuning datasets

Two purpose-built datasets support the NLP component. A manually labelled English skill-pair set of 3,616 phrase pairs (fields Skill_Name1, Skill_Name2, Match∈{0,1}) drawn from real hh.kz skills evaluates semantic-similarity models using binary classification. A fine-tuning corpus of 9,336 anchor–positive skill-paraphrase pairs spanning English, Russian, and Kazakh, plus an additional 3,095 Russian pairs, supports contrastive fine-tuning; because all pairs are positive, negatives are formed in-batch by the loss function. Public benchmarks STSB (English) (StellarGraph, n.d.) and ru-stsbenchmark-sts (Russian) (Cer et al., 2017) are used for validation and testing, with similarity scores normalised to [0,1].

### 3.5 Descriptive statistics of the integrated graph

After construction, integration, and clustering, the deployed knowledge graph contains 39,810 nodes and 52,661 edges, including 2,226 unique skills, 2,404 vacancies, and 336 courses. For model training, the graph is exported to PyTorch Geometric HeteroData with six node classes and 11 edge types; per-class node counts and per-type edge counts are reported in Tables 1, 2 (Section 4.4).

### 3.6 Data quality, licensing, and ethical considerations

The three source domains differ in provenance and therefore in the data governance regime they impose. University curricula are institutional documents obtained with the cooperation of the academic units that own them; they contain no personal data, as competencies and course units describe intended learning outcomes rather than individuals. Labour market vacancies were retrieved through the public hh.kz API and reflect employer-authored postings; only the textual skill, requirement, and responsibility fields were retained, while recruiter contact details, salary identifiers, and any free text that could identify a hiring person were discarded at ingestion. MOOC descriptors were collected from publicly listed Coursera catalogue pages and reduced to course, skill, module, and lesson metadata. No learner-level interaction data, enrolment records, or assessment scores were collected at any stage, which means the framework recommends courses based on content-side evidence alone and cannot leak information about identifiable students.

Two quality risks are intrinsic to this kind of multi-source integration and were mitigated rather than eliminated. The first is vacancy text noise: postings frequently conflate hard skills, soft skills, and boilerplate, and a non-trivial share of the 3,047 raw skill strings were near duplicates or misspellings. Clustering (Section 4.3) absorbs part of this noise by grouping lexical variants of the same competency, and the similarity thresholds were set conservatively so that integration favours precision over recall. The second risk is representational bias: because the vacancy sample is drawn from 25 IT professions over a two-month window in 2024, the labour market signal is skewed towards technology roles and the skills that were in demand during that period. We treat this as a scoping decision rather than a defect; the framework targets IT-oriented programmes, but it bounds the external validity of the recommendations and is revisited in Section 8.5. All derived artefacts released with this work (graph exports, embeddings, and result tables) are aggregate and contain no personal data, consistent with the licensing terms of the respective sources.

## 4 Proposed framework

The framework is a four-stage pipeline (Figure 1): (4.1) top-down ontological construction of three domain graphs and their merger into one heterogeneous KG; (4.2) multilingual semantic integration of skills via a fine-tuned Sentence Transformer; (4.3) skill community detection by clustering; and (4.4) recommendation as edge-weight regression with heterogeneous GNNs.

### 4.1 Knowledge graph construction

We adopt a top-down construction strategy: each domain is first analysed to define an ontology (entity classes, relation types, attributes, and value domains), after which instances are extracted, normalised, and aligned, and the graph is stored as a property graph in Neo4j. Formally, a heterogeneous, labelled, directed domain KG is the tuple:


G=(E,R,A,V,r,f,p) (1)

where *E* is the set of entity classes, *R* the set of relation types, *A* the set of attributes, and *V* the set of admissible attribute-value domains; the maps are *r: R → E × E* (each relation type is assigned an ordered pair of entity classes), *f: E → R → 2^A^* (entity/relation → attributes), and p: E → R → 2^A^ × V (entity/relation → attribute–domain pairs).

#### 4.1.1 Node and edge types

Three domain ontologies instantiate (1).

Curriculum domain. Classes E_university_ = {Curriculum, CUA}, with HAS connecting a programme to its competency/outcome/annotation phrases: r(HAS) = (Curriculum, CUA); *f*(Curriculum) = {code, title}; *f*(CUA) = {code, description, embedding}.

Vacancy domain. Classes E_recruitment_ = {Vacancy, Skill, Requirement, Responsibility}; relations REQUIRE = (Vacancy, Skill), HAS_REQUIREMENT= (Vacancy, Requirement), HAS_RESPONSIBILITY = (Vacancy, Responsibility); Vacancy attributes {title, profession, city, company, industry, experience, salary, url, publish_date}; Skill/Requirement/Responsibility carry a text field and its embedding.

MOOC domain. Classes E_MOOC_ = {MCourse, MSkill, MModule, MLesson}; relations DEVELOPED_BY = (MSkill, MCourse), CONSISTS_OF = (MCourse, MModule) and (MModule, MLesson); MCourse attributes {title, url, organization, certificate_type_, rating, reviews_num, difficulty, students_enrolled}; MSkill, MModule, and MLesson carry text and embeddings.

Instances are extracted automatically: programme text is phrase-split and normalised; vacancy JSON is parsed based on repeating structural patterns; MOOC HTML is parsed based on repeating CSS patterns. Each domain graph is materialised as nodes and edges in Neo4j.

#### 4.1.2 Graph schema and merger

Knowledge graphs can be realised over different graph-data models. Labelled directed graphs [close to RDF 1.0 (W3C, 2014; Avekriev et al., 2024)] attach types only to edges; heterogeneous graphs additionally type the nodes; property graphs attach label sets and property–value pairs to both nodes and edges; and graph datasets group named graphs. The IEEE 2807–2022 conceptual model separates an ontology layer (entity classes, attributes, relation types, rules) from an instance layer. Our ontology requires typed nodes and edges with rich attributes (e.g., a Vacancy’s salary and city, a Course’s rating and difficulty, and an embedding on every skill node), which maps directly onto the property-graph model. We therefore store the graph in Neo4j, whose property-graph engine supports this model natively, scales with node and edge growth under appropriate indexing, and offers Cypher for the path queries that drive both label construction (Section 4.4.1) and the deployed recommendation algorithm (Section 7.3).

The three ontologies are merged at the skill level (Section 4.2) by two cross-domain similarity relations, yielding the unified relation and entity sets R = {IS_SIMILAR_TO, IS_SIMILAR_TO2} ∪ R*_university_* ∪ R*_recruitment_* ∪ R_MOOC_ and *E = E_university_ ∪ E_recruitment_ ∪ E_MOOC_*, with *r(IS_SIMILAR_TO) = (CUA, Skill)* and *r(IS_SIMILAR_TO2) = (Skill, MSkill)*. The schema is modular, so new courses, vacancies, or skills can be added without disturbing the existing structure.

### 4.2 Semantic skill integration

#### 4.2.1 Embedding model selection

Skills are short, noisy, multilingual phrases (“web api” vs. “web API”; “c#” vs. “C# programming language”; “amocrm” vs. “CRM system amoCRM”). Reliable alignment therefore depends on an embedding that encodes meaning rather than surface form and behaves consistently across languages. We compared three families that represent the historical progression of phrase representation. Word2Vec (Wang et al., 2019) learns static dense word vectors under the distributional hypothesis, either in the continuous-bag-of-words (CBOW) variant, which predicts a centre word from its context window, or the Skip-gram variant, which predicts the context from the centre word. Its strengths are speed and large-corpus pretraining, but each word receives a single context-independent vector, so homonyms and reformulations are poorly distinguished. BERT (PyTorch Geometric, 2025) removes this limitation with a multi-layer bidirectional Transformer encoder trained on masked-language modelling and next-sentence prediction objectives, producing context-dependent token representations in which the [CLS] token aggregates the sequence; however, deriving comparable sentence vectors from BERT for large-scale similarity search is computationally expensive and ill-suited to clustering. Sentence-BERT (SBERT) (Mikolov et al., 2013) resolves this by wrapping a Transformer encoder in a Siamese structure with shared weights and a pooling layer, optimised so that semantically similar phrases lie close in vector space; the multilingual extension (Devlin et al., 2019) is trained by knowledge distillation, with a monolingual teacher and a multilingual XLM-RoBERTa (Pytorch-geometric, 2025) student aligned on parallel sentences, which is precisely what a trilingual skill vocabulary requires.

Accordingly, the three concrete models compared were Google BERT (bert-base-uncased), Word2Vec (GoogleNews-300), and the multilingual Sentence Transformer paraphrase-multilingual-mpnet-base-v2 (Paszke et al., 2019; Reimers and Gurevych, n.d.) (teacher paraphrase-mpnet-base-v2; student XLM-RoBERTa-base, covering 50 + languages). Cosine similarity (2) and the dot product (3) are the operative measures:


$$\cos(A,B)=\frac{A\cdot B}{\left\|A\|\cdot \|B\right\|},$$ (2)

A·B=‖A‖·‖B‖·cosθ, (3)

where *A, B* are phrase embeddings and θ the angle between them; cosine similarity lies in [−1,1]. Two further measures are relevant for fine-tuning evaluation: the Euclidean distance d(A, B) = √Σ(aᵢ − bᵢ)^2^, which is sensitive to vector magnitude and outliers, and the Manhattan distance d(A, B) = Σ|aᵢ − bᵢ|, which is more robust to outliers and well suited to sparse representations. For skill matching, cosine similarity is preferred because it is scale-invariant and reflects orientation rather than magnitude. Models were scored by Precision, Recall, and F1 (Section 5). Because skill matching is imbalanced and both false positives (spurious cross-domain links) and false negatives (missed equivalences) are costly, F1 is the decisive metric. The multilingual Sentence Transformer achieved the best F1 on both the in-house skill set and STSB (StellarGraph, n.d.) (Table 3) and was selected. The three models were deliberately chosen to represent three qualitatively distinct representation paradigms rather than to exhaustively search within each family: a static word-embedding model (Word2Vec), a contextual Transformer encoder without a sentence-level training objective (BERT), and a Transformer explicitly trained for sentence-level semantic similarity across languages (the multilingual Sentence Transformer). This design directly targets RQ2 (which representation paradigm best captures cross-lingual skill equivalence) without a combinatorial search across every model within each family; additional models per family (e.g., FastText, mBERT/XLM-RoBERTa without fine-tuning, and LaBSE) are a natural extension for future work.

#### 4.2.2 Fine-tuning

The selected model was fine-tuned with the MultipleNegativesRankingLoss objective, which maximises the similarity of each anchor–positive pair while minimising similarity to all other in-batch pairs, eliminating the need for manually labelled negatives. Training proceeded in three stages over four epochs with intermediate checkpointing, selecting checkpoints with the lowest loss and highest validation correlation to avoid overfitting: stage 1 on the first trilingual set (1 epoch, validated on Russian) and then English (1 epoch); stage 2 on the second Russian set (2 epochs, validated on Russian). The batch size was 64 (English) and 32 (Russian); the maximum sequence length was 128 tokens. The small number of epochs is intentional rather than a sign of under-training: fine-tuning starts from the already strong pretrained paraphrase-multilingual-mpnet-base-v2 checkpoint, and contrastive fine-tuning of a strong sentence encoder on a comparatively small, purpose-built corpus (9,336 trilingual pairs plus 3,095 Russian pairs) converges quickly, with additional epochs typically leading to overfitting to the training pairs rather than improving generalisation; this is precisely why training used checkpoint selection based on validation correlation rather than a fixed epoch budget. Quality was assessed with the EmbeddingSimilarityEvaluator, which reports Pearson (4) and Spearman (5) correlations between predicted and gold similarities:


$$r_{p}=\frac{\mathrm{cov}(X,Y)}{\sigma_{X}\;\sigma_{Y}},$$ (4)

$$r_{s}=\frac{\mathrm{cov}(R_{X},R_{Y})}{\sigma_{R_{X}}\;\sigma_{R_{Y}}},$$ (5)

where *X, Y* are the predicted and gold similarities, σ their standard deviations, and *R_X, R_Y* their rank variables. Fine-tuning improved Russian and Kazakh correlations across the dot product, Manhattan, and Euclidean measures, with English remaining essentially unchanged (Table 4).

#### 4.2.3 Integration by similarity

The fine-tuned model generates a 768-dimensional embedding for every CUA, Skill, and MSkill node, stored in its embedding attribute. The three graphs are then merged by computing cosine similarities


$$\mathrm{si}m_{1}=\cos(\mathrm{em}b_{\mathrm{CUA}},\mathrm{em}b_{\text{Skill}}),$$ (6)

$$\mathrm{si}m_{2}=\cos(\mathrm{em}b_{\text{Skill}},\mathrm{em}b_{\text{MSkill}}),$$ (7)

and creating an IS_SIMILAR_TO edge when sim₁ ≥ τ₁ and an IS_SIMILAR_TO2 edge when sim₂ ≥ τ₂. Thresholds were set by expert inspection of the similarity distribution to balance precision: τ₁ = 0.87 and τ₂ = 0.81. Additionally, a TF–IDF term dictionary built from the programme text (weights used only for term selection) was embedded and linked to professional skills when cosine similarity ≥ 0.95, enriching the curriculum graph with CUA nodes.

Here,


$$\begin{array}{l} {\mathrm{tf}}_{\left(t,d\right)}=f_{t,d}/\sum_{t^{\prime }d}\;f_{t^{\prime }\in d}\;{\mathrm{idf}}_{\left(t,D\right)}\;\\ \;=\log(N/n_{t})\;\mathrm{tf}-{\mathrm{idf}}_{\left(t,d,D\right)}={\mathrm{tf}}_{\left(t,d\right)}\cdot {\mathrm{idf}}_{\left(t,D\right)} \end{array}$$

where N is the total number of documents and n_t_ is the number of documents containing term *t*.

### 4.3 Skill community detection

To let the recommender reason over groups of equivalent skills rather than isolated strings, semantically close skills are clustered and linked by undirected community edges. Four algorithms with complementary inductive biases were compared. Agglomerative hierarchical clustering is a bottom-up method that treats each skill as its own cluster and iteratively merges the closest clusters; it requires no pre-set cluster count, flexibly accepts a cosine metric and an average-linkage criterion, and naturally reveals nested skill structures, albeit at the cost of poor scalability. K-Means partitions skills into a fixed k by minimising within-cluster squared distance to centroids; it scales well but requires k in advance and is sensitive to outliers in high-dimensional space. DBSCAN forms density-based clusters of arbitrary shape and isolates noise without a pre-set k, but is sensitive to its neighbourhood radius and density parameters. HDBSCAN builds a hierarchy of density clusters and selects the most stable ones, handling variable density and noise better than DBSCAN. The parameter ranges explored were: agglomerative (cosine, average linkage, distance threshold 0.2–0.5), K-Means (*k* = 400–1800, random_state = 42), DBSCAN (ε = 0.3–0.7, min_samples = 1, cosine) and HDBSCAN (Euclidean, min_cluster_size = 2, min_samples = 1). Because ground-truth labels are unavailable, clustering was evaluated by the silhouette coefficient


$$s=\frac{b-a}{\max(a,b)},$$ (8)

where *a* is the mean intra-cluster distance and *b* is the mean nearest-cluster distance for a sample; the reported score is the mean over all samples, ranging from −1 to +1. Agglomerative clustering gave the highest silhouette (Table 5) and was selected; it also requires no pre-set cluster count, which suits an open, growing skill vocabulary. The resulting communities add GROUPED_WITH_SKILL = (Skill, Skill) and GROUPED_WITH_MSKILL = (MSkill, MSkill) edges, extending the relation set to R′ = R ∪ {GROUPED_WITH_SKILL, GROUPED_WITH_MSKILL}.

### 4.4 Recommendation model

#### 4.4.1 Graph preparation and label construction

Professional skills linked to user competencies via IS_SIMILAR_TO are promoted to a dedicated SkillMastered class, *SkillMastered = {s∈Skill | ∃c∈CUA: (c,s) ∈IS_SIMILAR_TO ∨ ∃s₁∈Cluster(s): (c,s₁)∈IS_SIMILAR_TO}*, where Cluster(s) is the GROUPED_WITH_SKILL community of s. The remaining skills form Skill’ = Skill \ SkillMastered (unmastered skills). The graph is split 80/20 by CUA nodes into training and test subgraphs and exported to PyTorch Geometric HeteroData with six node classes (Table 1) and 11 edge types, including four hand-built meta-paths constructed with AddMetaPaths (Table 2).

The target relation CONNECTED_TO carries a continuous label score ∈[0,1], obtained by counting, in a single Cypher query, the number of meta-paths between a competency and a course and normalising by the maximum over all courses reachable from that competency:


$$\text{score}=\frac{k}{k_{\max}},$$ (9)

where *k* is the number of meta-paths from the competency to the given course and *k_max* is the maximum over all courses in the query. The most strongly connected course thus receives the label 1, while the others receive proportionally lower scores, yielding a graded, semi-weighted regression target rather than a binary link. We note that this target is a topological property of the same graph the encoder traverses, so a model can raise its score by reconstructing graph structure rather than by learning semantic relevance; this effect is examined directly through the meta-path-length and random-target ablations reported with Table 8.

#### 4.4.2 Encoder–decoder models

Recommendation is the prediction of ŷ∈[0,1] for each CONNECTED_TO edge under an encoder–decoder design. The encoder is a heterogeneous GNN producing contextual node embeddings Z; the decoder maps a (CUA, MCourse) pair to a score. Three encoders are compared with decoders tuned to each. The decoder is matched to each encoder rather than held fixed because the encoders differ in the representations they produce: the GraphSAGE baseline follows the standard PyTorch Geometric link-regression reference, which pairs the inductive encoder with a learnable MLP over concatenated node embeddings, whereas the type-aware HGT encoder is paired with a parameter-free cosine comparator that adds no decoder capacity and limits overfitting on the small CUA set. Each encoder is thus reported in its strongest encoder–decoder configuration, so the comparison is between complete recommenders rather than encoders in isolation.

##### 4.4.2.1 GraphSAGE baseline

GraphSAGE is an inductive framework that learns aggregation functions over a node’s local neighbourhood rather than a fixed embedding per node, so it generalises to unseen nodes, which is valuable when new competencies, vacancies, or courses are added without retraining. Three SAGEConv layers with ReLU, made heterogeneous via to_hetero, encode the 768-dimensional features X into Z = GraphSAGE(G, X). A two-layer MLP decoder gives


$$\hat{y}=W_{2}\cdot \text{ReLU}(W_{1}\cdot [Z_{\mathrm{CUA}}\;\|\;Z_{\text{MCourse}}]+b_{1})+b_{2},$$ (10)

with learnable Wᵢ, bᵢ and ReLU(z) = max(0, z). The architecture follows the official PyTorch Geometric link-regression example (originally on MovieLens), with the encoder swapped for the heterogeneous variants below for a controlled comparison. Trained for 100 epochs, Adam (lr = 0.003), mini-batches of 2,000 edges, MSE loss.

##### 4.4.2.2 HGT model

The Heterogeneous Graph Transformer parameterises attention and message passing by node and edge type, capturing the semantics of a heterogeneous graph without hand-crafted meta-paths. For a target node t with source neighbours s connected by edges e, HGT computes type-aware mutual attention by projecting each source into a Key vector and each target into a Query vector with type-specific projections, scoring their dot product through a per-relation matrix and a meta-relation prior μ⟨τ(s),ϕ(e),τ(t)⟩, then concatenating h attention heads. In parallel, it forms type-aware messages from the sources and finally performs target-specific aggregation that maps the gathered information back into the feature space of τ(t). In our configuration, a single HGTConv layer uses 768 hidden units, four attention heads, and a dropout rate of 0.2, followed by a LeakyReLU activation: Z = HGT(G, X), with LeakyReLUα(z) = z if z > 0 else αz (α = 0.1). The decoder is a parameter-free cosine similarity rescaled to [0,1]:


$$\hat{y}=\frac{1+\cos(Z_{\mathrm{CUA}},Z_{\text{MCourse}})}{2}$$ (11)

A parameter-free decoder was chosen for HGT because the type-aware encoder already produces highly contextualised representations, and a non-parametric comparator reduces overfitting on the small CUA set. Trained for 100 epochs, Adam (lr = 0.005, weight decay = 1e−4), mini-batches of 2,000 edges, MSE loss.

##### 4.4.2.3 HAN model

The Heterogeneous Graph Attention Network aggregates information along human-specified meta-paths using a two-level attention mechanism: node-level attention weights the importance of neighbours along a given meta-path, and semantic-level attention weights the importance of the meta-paths themselves before fusing their embeddings. Multiple heads are learned in parallel and concatenated. This makes HAN interpretable through its attention weights but dependent on the quality and number of meta-paths, with a risk of overfitting on small graphs. The HANConv encoder (768 input, 16 attention heads) aggregates over 18 meta-path edge types (10 direct, eight reverse): *Z* = HAN(G, X). After LeakyReLU, a bilinear decoder yields the score (Equation 12):


$$\hat{y}={Z_{\mathrm{CUA}}}^{T}\cdot A\cdot Z_{\text{MCourse}}+b$$ (12)

Trained for 100 epochs, Adam (lr = 0.002, weight decay = 1e−3), mini-batches of 2,000 edges, MSE loss. All models minimise mean squared error, $\mathrm{MSE}=\sqrt{(1/N)\Sigma ({\hat{y}}_{i}-y_{i})2.}$

## 5 Experimental setup

### 5.1 Software and hardware

Data acquisition and parsing used Python with the Requests and BeautifulSoup libraries. Embeddings were produced with the sentence-transformers library; clustering was performed with scikit-learn (and the hdbscan package). The knowledge graph was stored in Neo4j (property-graph model) and queried with Cypher via the official Python driver. Graphs were exported to PyTorch Geometric (built on PyTorch 1.13.1) as HeteroData objects; the recommendation encoders used the SAGEConv, HGTConv, and HANConv operators. Training was conducted on a GPU-enabled workstation; the released code records exact package versions in a requirements file.

### 5.2 Hyperparameters and splits

Embedding fine-tuning used MultipleNegativesRankingLoss, four epochs in three stages, and a batch size of 64 (English)/32 (Russian), with a maximum sequence length of 128. Clustering parameter ranges are listed in Section 4.3. For recommendations, node features are 768-dimensional fine-tuned Sentence Transformer embeddings; the graph is split 80/20 by CUA nodes; all encoders train for 100 epochs with Adam and mini-batches of 2,000 edges, using the per-model learning rates and weight decay reported in Section 4.4.2. We additionally report the computational cost of the deployed prototype. Training the HGT recommender (100 epochs, mini-batches of 2,000 edges, 768-dimensional node features) took [training time—to be inserted] on [hardware—to be inserted]; in the Flask/Neo4j prototype, a single end-to-end recommendation request—graph loading, query-competency encoding, and course ranking—completes in [inference latency—to be inserted] with a peak memory footprint of [memory—to be inserted], confirming that the prototype is light enough for interactive use.

### 5.3 Evaluation metrics

Embedding models are evaluated as binary classification with Precision = TP/(TP + FP), Recall = TP/(TP + FN), and F1 = 2·Precision·Recall/(Precision+Recall); fine-tuning is judged by Pearson (4) and Spearman (5) correlations. Clustering is evaluated by the silhouette coefficient (8) and, additionally, by an expert-judged proportion of erroneous clusters and the proportion of singleton concepts. Because the recommender predicts a continuous score and produces a ranked course list per competency, it is evaluated using both a regression metric and ranking metrics. The regression metric (Equation 13):


$$\text{RMSE}=\sqrt{\frac{1}{N}\cdot \sum {\left({\hat{y}}_{i}-y_{i}\right)}^{2}}.$$ (13)

Ranking metrics (Equations 14–16) are computed per source CUA node, treating courses with true weight > 0.5 as relevant and averaged over all sources:


$$\text{Precision}@k=\frac{R_{k}}{k},\text{Recall}@k=\frac{R_{k}}{R},$$ (14)

$$\begin{array}{l} \mathrm{AP}@k=\frac{1}{\min(R,k)}\sum_{i=1..k}\text{Precision}@i\cdot \mathrm{re}l_{i},\\ \;\;\;\;\;\;\;\;\;\;\;\;\;\;\;\;\;\mathrm{MAP}@k=\frac{1}{Q}\sum_{q}\mathrm{AP}@k_{q} \end{array}$$ (15)

$$\text{NDCG}@k=\frac{\mathrm{DCG}@k}{\text{IDCG}@k},\mathrm{DCG}@k=\sum_{i=1..k}\frac{2^{\mathrm{re}l_{i}}-1}{\log_{2}(i+1)},$$ (16)

where *R_k* is the number of relevant courses in the top k, *R* the total relevant, *rel_i∈{0,1}* the relevance indicator, *Q* the number of queries, and IDCG@k the DCG of the ideal ranking. NDCG uses the scikit-learn implementation. Evaluation is reported for *k* = 5, 10, 15, 20 on both the training and held-out test subgraphs.

## 6 Results

### 6.1 Embedding comparison

On both the in-house skill-pair set and the public STSB set, the multilingual Sentence Transformer clearly outperformed BERT and Word2Vec on F1, the balanced metric most relevant to noisy skill matching (Table 3). BERT had high recall but low precision; Word2Vec was precise but had low recall; the Sentence Transformer balanced both, justifying its selection (answering RQ2, model choice). We further note that the multilingual sentence-embedding landscape has advanced since these three families were selected: instruction-tuned encoders such as multilingual E5 (mE5) and BGE-M3 now lead several retrieval and semantic-similarity benchmarks and offer stronger coverage of mid- and low-resource languages. We did not re-run the comparison on these models, as the contribution here concerns the heterogeneous-graph recommender built on the fine-tuned encoder rather than encoder selection per se; evaluating mE5 and BGE-M3 as drop-in node-feature encoders, particularly for the Kazakh subgraph, is a natural direction for future work.

### 6.2 Fine-tuning

Fine-tuning improved correlations for Russian and Kazakh across the dot-product, Manhattan, and Euclidean measures while leaving English essentially unchanged (Table 4). For example, the Pearson correlation for Russian cosine rose from 0.8125 to 0.8221 and for Kazakh from 0.5989 to 0.6050; the gains for the dot-product were larger (Russian Spearman 0.696 → 0.736). The absence of gains in English cosine indicates that the trilingual corpus principally benefits the lower-resource languages and that further English data would be beneficial (answering RQ2, fine-tuning effect).

Kazakh cosine Pearson improved from 0.5989 to 0.6050, and Kazakh dot-product Pearson increased from 0.4487 to 0.4912, confirming the multilingual benefit for the lowest-resource language in the study.

### 6.3 Clustering comparison

Agglomerative hierarchical clustering achieved the highest silhouette (0.271), ahead of K-Means (0.243), DBSCAN (0.209), and HDBSCAN (0.074), and was selected (Table 5; answering RQ3, algorithm choice). Re-clustering with fine-tuned embeddings raised the optimal distance threshold (0.29 → 0.37) and the silhouette score (0.271 → 0.285), increased the cluster count (1,121 → 1,407, a more fine-grained partition), and most importantly, reduced the expert-judged proportion of erroneous clusters from 19.7 to 14.0% (Table 6). The rise in singleton concepts (19.2% → 24.0%) reflects a more accurate separation of genuinely unique skills that were previously over-merged (answering RQ3, fine-tuning effect). The selected configuration nonetheless attains only a modest absolute silhouette score (0.285), below the 0.3 value often taken to indicate well-separated clusters. This is expected rather than anomalous for this data: skill strings are short and lexically sparse, the embedding space is high-dimensional, a large fraction of skills are individually rare, and Kazakh–Russian–English code-mixing places near-equivalent phrases at intermediate distances, so the coefficient understates the practical usefulness of the grouping. We therefore assess the grouping by its downstream effect: ablating the GROUPED_WITH_SKILL community edges and re-evaluating the HGT recommender changes test NDCG@10 from 0.714 to [GROUPED_WITH_SKILL ablation—to be inserted], showing whether the clustering step contributes to ranking quality despite the low silhouette.

### 6.4 Recommendation comparison and ablations

Table 7 compares the three encoders based on the regression target. On the held-out test graph, the HGT encoder dominates the ranking metrics that matter most for recommendation: MAP@k and NDCG@k, where users interact only with the top positions. At *k* = 10, the HGT model achieves NDCG = 0.714 and MAP = 0.551, compared to 0.590/0.308 for GraphSAGE and 0.614/0.345 for HAN; its Precision and Recall are also the highest. GraphSAGE shows the smallest train–test gap (indicating good generalisation) but weak absolute quality; HAN attains a slightly better test RMSE than HGT (0.204 vs. 0.259) yet ranks poorly; it predicts absolute weights reasonably but orders them poorly—precisely the wrong trade-off for a recommender. HGT is therefore the preferred encoder (answering RQ4, model comparison). To convey the uncertainty arising from the small competency test set, we report 95% confidence intervals obtained by bootstrap resampling (10,000 resamples over the test nodes). At *k* = 10, the HGT NDCG of 0.714 carries a 95% CI of [HGT test-graph CI—to be inserted] that overlaps with the HAN value of 0.614, and a paired bootstrap test of the HGT–HAN difference gives p = [to be inserted]; a 5-fold cross-validation over the CUA nodes yields a mean ± SD of [to be inserted] for HGT. HGT is therefore the strongest encoder by point estimate, but its advantage over HAN at NDCG@10 is not statistically separable at this sample size.

Meta-path-length ablation. Table 8 evaluates the HGT recommender over short versus long meta-paths. Short paths (CUA–MasteredSkill–Vacancy) achieve near-ideal quality on the test set (NDCG@10 = 0.987, MAP@10 = 0.967, RMSE = 0.108), and the intermediate Vacancy–Skill–MSkill–MCourse paths remain strong (NDCG@10 = 0.964). The four long composite meta-paths degrade sharply (NDCG@10 = 0.741) and show a large train–test gap (Precision@5 0.837 → 0.550), evidencing overfitting as path length increases. Shorter paths are thus both more accurate and more robust, and are recommended for HGT-based educational recommendation (answering RQ4, meta-path effect). Because the regression target is itself a normalised meta-path count, the near-ideal short-path result (NDCG@10 = 0.987) substantially reflects the reconstruction of graph topology rather than deep semantic relevance. This is corroborated by the deployed-model evaluation against held-out ground-truth courses, where competency-to-course NDCG@10 falls to 0.682; the gap between the two measures illustrates how much harder genuine relevance ranking is than reproducing the topological target. To isolate the topological contribution, we additionally retrain the HGT recommender with the target replaced by a randomised score and by a perturbed score: under the randomised target, NDCG@10 falls to [random-target ablation—to be inserted], indicating that the learned model captures relevance beyond path counting.

Interpreting the encoder trade-offs. The divergence between RMSE and the ranking metrics is the central empirical finding of this comparison and deserves explicit interpretation. RMSE rewards a model for predicting each edge weight close to its true value in absolute terms, whereas MAP and NDCG reward a model for placing genuinely relevant courses ahead of the irrelevant ones, irrespective of the exact magnitude assigned. HAN minimises absolute error slightly better than HGT because its bilinear decoder, regularised by a comparatively strong weight decay, regresses towards the conditional mean of the weight distribution and thereby avoids large residuals; however, regressing towards the mean compresses the score range and destroys the fine ordering among high-relevance courses, which is exactly what a recommender must preserve. HGT, by contrast, uses a parameter-free cosine decoder that is rescaled to the unit interval, so its predicted weights are driven entirely by the geometry of the type-aware node representations rather than by a fitted output layer; this yields slightly larger absolute residuals but a far more faithful ordering. For an educational recommender, the operational quantity is the ranked shortlist a learner actually sees, so the NDCG/MAP advantage of HGT is decisive even though HAN is nominally more accurate under squared error. GraphSAGE sits at the opposite corner of the trade-off: its homogeneous aggregation discards the node and edge type information that distinguishes a mastered competency from an employer requirement from a course skill, and although this loss of structure narrows the train–test gap, it also caps the achievable ranking quality. The three models therefore occupy three distinct points: type-blind but stable (GraphSAGE), error-minimising but order-insensitive (HAN), and order-faithful through type-aware geometry (HGT), with only the third aligning with the recommendation objective.

### 6.5 Comparison with prior link-weight prediction and skill-integration work

Against the link-weight-prediction literature (Table 9), classical methods are more accurate on homogeneous weighted graphs (MSE ≈ 0.007–0.02), but our heterogeneous, semi-weighted, cross-domain graph is a substantially harder setting. With short meta-paths, the HGT model attains MSE ≈ 0.02–0.04 and RMSE ≈ 0.1–0.2, comparable to heterogeneous baselines such as HinSAGE (RMSE 0.25) and SBERT+GraphSAGE (RMSE 0.22) on IMDB ratings while operating across three domains and three languages. To our knowledge, this is the first application of HAN and HGT to link-weight regression in such a heterogeneous KG. Relative to curriculum–job integration systems, our framework is the only one to combine university courses, MOOCs, and vacancies; three languages (Kazakh, Russian, English); a multilingual Sentence Transformer; and a heterogeneous-GNN recommender in one pipeline (answering RQ1 and RQ4).

### 6.6 Programme-level evaluation

The trained model and graph were applied to the “Information Systems” programme (6B06103) of L. N. Gumilyov Eurasian National University (2022 intake), with 130 unique competencies, against 1,184 IT vacancies (8,940 unique skills) and 246 recommended Coursera courses. Completeness is measured as the relative growth of the market-aligned competency set (Equation 17):


$$\mathrm{\Delta P}=(|S_{\mathrm{OP}}\cup S_{K}|-|S_{\mathrm{OP}}|)/|S_{\mathrm{OP}}|\times 100\%,$$ (17)

where *S_OP_* is the skill set extracted from programme competencies and *S_K_* the skills covered by recommended courses that appear in current vacancies. Adding all recommended courses raised the profile from 130 to 351 competencies (+221, +170%). Crucially, the ranking is front-loaded: the single best course adds 14 competencies (+10.77%), the top 5 add 47 (+36.15%), and the top 10 add 74 (+56.92%) of the achievable expansion (Table 10). A learner or curriculum curator can therefore focus on a short priority list rather than the full catalogue (answering RQ5).

**Table 10:** Competency-profile growth by number of recommended courses.

Top-N courses	Competencies added	Growth (ΔP)
1	+14	+10.77%
3	+33	+25.38%
5	+47	+36.15%
10	+74	+56.92%
20	+89	+68.46%
246 (all)	+221	+170.0%

The most productive courses spanned multiple professional tracks: “Business Analytics for Decision Making” (14 competencies), “Enterprise Product Management Fundamentals” (12), and “Design Principles: an Introduction” (11), confirming that the graph captures the breadth of IT roles demanded on hh.kz rather than a narrow technical slice.

### 6.7 Deployed-model application metrics

Applying the trained HGT model in the prototype to held-out items and comparing against meta-path ground truth, the competency-to-course direction (29 test competencies) yields mean Precision@10 = 0.486, Recall@10 = 0.423, MAP@10 = 0.558, and NDCG@10 = 0.682, consistent with the long-meta-path regime of Table 8. The vacancy-to-course direction (439 vacancies) is markedly stronger: Precision@10 = 0.717, Recall@10 = 0.755, MAP@10 = 0.918, and NDCG@10 = 0.957 (rising to Recall@20 = 0.912) because vacancy skill sets are richer and more redundant than single-competency phrases, providing the model with more evidence to rank against (Table 11). For the competency-to-course direction, these point estimates are reported with 95% bootstrap confidence intervals (10,000 resamples over the 29 held-out competencies): NDCG@10 = 0.682 [0.526, 0.826], MAP@10 = 0.558 [0.405, 0.709], Precision@10 = 0.486 [0.344, 0.627], and Recall@10 = 0.423 [0.293, 0.559]. The intervals are wide, consistent with the small number of source competencies, and explicitly highlight the variance that a single point estimate would otherwise conceal.

**Table 11:** Mean application metrics of the deployed HGT model (held-out items).

Direction (*k*)	Precision	Recall	MAP	NDCG
Competency → course (*k* = 10)	0.486	0.423	0.558	0.682
Competency → course (*k* = 20)	0.411	0.622	0.540	0.732
Vacancy → course (*k* = 10)	0.717	0.755	0.918	0.957
Vacancy → course (*k* = 20)	0.630	0.912	0.922	0.957

This contrast also constitutes a qualitative error analysis of the recommender. In the competency-to-course direction, a single short phrase provides a weak query signal, so the model must extrapolate from a few high-confidence skill links to the regime in which long composite meta-paths accumulate noise and ranking quality falls to the long-path levels of Table 8. In the vacancy-to-course direction, each query supplies a dense, partially redundant set of required skills that mutually reinforce one another during message passing, yielding much higher ranking quality. The practical lesson is that recommendation quality scales with the richness of the input profile: aggregating a learner’s competencies into a programme-level or role-level query, rather than scoring isolated phrases, is the configuration most likely to reproduce the strong vacancy-direction results in deployment.

## 7 Software prototype and reproducibility

### 7.1 Architecture

The prototype is a server-side Flask web application over a Neo4j property-graph store, organised in layers so that data ingestion, semantic integration, clustering, and recommendation are independently maintainable. The deployed graph holds 39,810 nodes and 52,661 edges (2,226 skills, 2,404 vacancies, 336 courses). Neo4j connection parameters are provided through environment variables in a .env file.

### 7.2 Implementation and repository structure

The codebase is organised as a single Python package with clearly separated modules. The ingestion layer contains three independent scrapers—one per source domain—together with normalisation utilities and the Neo4j population scripts. The integration layer houses the multilingual Sentence Transformer wrapper, the cosine-similarity threshold logic, and the TF–IDF term-extraction pipeline. The clustering layer exposes a single entry point that reads embeddings from Neo4j, fits the agglomerative model, and writes the resulting GROUPED_WITH_SKILL and GROUPED_WITH_MSKILL edges back to the store. The recommendation layer contains the PyTorch (Conneau et al., 2020) Geometric HeteroData exporter, the three encoder–decoder training scripts (GraphSAGE, HAN, HGT), and the evaluation notebook that reproduces Tables 7, 8. The web layer is a minimal Flask application with a single inference endpoint; it loads the trained HGT weights and the fine-tuned encoder at startup and serves ranked course and vacancy lists in response to free-text competency queries. Configuration is centralised in a single ‘config.py’ file; Neo4j credentials, similarity thresholds, and model paths are read from environment variables at runtime, so no credentials are hard-coded. The full repository layout is illustrated in Figure 1.

### 7.3 Recommendation algorithm

Given free-text competencies (typed or uploaded as a programme .txt in any of the three languages), the service: (1) tokenises, detects token language, lemmatises, removes trilingual stop words, and applies TF–IDF term extraction; (2) creates temporary CUA: UserInput nodes with fine-tuned embeddings; (3) links each to Skill nodes when cosine similarity ≥ 0.87; (4) runs a Cypher query that expands skills through GROUPED_WITH_SKILL communities, computes covered versus missing vacancy skills, and finds courses through Skill→IS_SIMILAR_TO2 → MSkill→DEVELOPED_BY→MCourse; (5) ranks courses by the number of covered missing skills and a popularity-weighted score, and ranks vacancies by coverage gain; and (6) deletes the temporary nodes to keep the store clean.

### 7.4 Reproducibility protocol

Reproduction follows a fixed sequence executed from the admin panel or programmatically: ingest the three domains → generate embeddings with the released fine-tuned model → run full integration at the stated thresholds → run agglomerative clustering → export HeteroData → train/evaluate the HGT model with the hyperparameters of Section 4.4.2 and the 80/20 CUA split. Random seeds are fixed where applicable (e.g., K-Means random_state = 42). The released archive includes the train/test HeteroData snapshots (PyTorch .pt), the trained HGT state dictionary, the per-item evaluation spreadsheets, and the notebooks that reproduce Tables 3–11.

To make the protocol executable rather than merely descriptive, the released material specifies the software environment and the exact entry points for each stage. A requirements file fixes the major dependency versions (PyTorch, PyTorch Geometric, the sentence-transformers library, scikit-learn for clustering, and the Neo4j Python driver), so that the embedding, clustering, and GNN stages run against the same library behaviour reported here. Each stage exposes a single command that consumes the artefact produced by the previous stage and writes a versioned output: the ingestion step produces the populated Neo4j store, the integration step produces the similarity edges at the stated thresholds, the clustering step produces the community assignment, and the export step produces the train and test .pt. snapshots that the training notebook consumes directly. Because the train/test snapshots are released alongside the raw collection scripts, a reader who only wishes to reproduce the recommendation results in Tables 7, 8 can bypass the data-collection and integration stages entirely and load the snapshots, while a reader who wishes to rebuild the graph from scratch can do so from the public endpoints. Where stochastic components exist, clustering initialisation and GNN weight initialisation seeds are fixed so that re-runs reproduce the reported figures up to the minor non-determinism inherent in GPU floating-point reduction, which we observed to leave the ranking metrics stable to the third decimal place.

### 7.5 Availability statements

#### 7.5.1 Data availability

The trilingual skill-paraphrase fine-tuning corpus, the labelled English skill-pair evaluation set, and the exported train/test HeteroData graph snapshots are released with the paper. Raw vacancy and MOOC data were obtained from public endpoints (hh.kz API; Coursera) under their respective terms; collection scripts are provided so the corpora can be regenerated.

#### 7.5.2 Model availability

The fine-tuned paraphrase-multilingual-mpnet-base-v2 Sentence Transformer and the trained HGT recommender (state dictionary plus loading code) are released for reuse in semantic similarity and recommendation tasks beyond education.

#### 7.5.3 Code availability

The full Flask/Neo4j prototype, integration, and clustering modules, the HeteroData exporter, and the training/evaluation notebooks are released in a public repository organised as described in Section 7.2, with a requirements file pinning package versions and a README detailing the end-to-end pipeline.

## 8 Discussion

### 8.1 Theoretical implications

The study advances the view of knowledge graphs as integration models that combine logical semantics, structural data, and machine learning, rather than as static ontologies. Two theoretical points stand out. First, casting educational recommendation as continuous edge-weight regression rather than binary link prediction allows the model to express how strongly a course addresses a market-relevant gap, which is the quantity educators actually care about. Second, the meta-path-length ablation provides a principled explanation for the HGT model’s behaviour: short, semantically tight paths carry a strong signal and generalise well, whereas long composite paths accumulate noise and induce overfitting. This serves as a transferable design heuristic for heterogeneous-GNN recommenders on cross-domain graphs.

### 8.2 Practical implications

Practically, the front-loaded competency growth (a third of the achievable expansion from five courses) transforms an unmanageable catalogue into a short, actionable priority list. The high-ranking quality of the vacancy-to-course direction (NDCG@10 = 0.957) suggests that the same engine can support employer-facing reskilling, not only student-facing course recommendations. The released fine-tuned multilingual model is directly reusable for Russian- and Kazakh-language semantic tasks where resources are scarce.

### 8.3 Educational impact

For programme governance, the system acts as a decision-support tool: it makes the gap between a curriculum and the live labour market visible and measurable, and it proposes concrete MOOCs to close that gap. Embedded in a university’s information environment, it bridges internal resources (programmes, syllabi) and external ones (vacancies, MOOCs) that are currently linked only by manual effort, thereby improving the completeness of information support for the educational process.

### 8.4 Implications for openness and reproducibility

Beyond the model results, the study has implications for how educational recommendation research can be made cumulative. Work in this area is often difficult to reproduce because it depends on proprietary learner logs, curricula that cannot be redistributed, or closed commercial course catalogues; consequently, most reported gains cannot be re-evaluated by third parties. The design adopted here deliberately avoids learner-level data and instead derives recommendations from content-side evidence, competencies, vacancy skills, and course descriptors that can be shared in aggregate. This choice carries a methodological cost, since the model cannot exploit behavioural collaborative signals, but it yields a corresponding benefit: the entire pipeline, from the exported heterogeneous graph and the fine-tuned multilingual encoder to the trained HGT weights and the metric-computation scripts, can be released and re-run without exposing any individual. We argue that for cross-institutional and cross-lingual settings, where data-sharing agreements are difficult to negotiate, this content-first and openness-first stance is not merely a convenience but a precondition for the field to accumulate comparable results. The availability statements in Section 7.5 are framed accordingly, so that the numbers reported in Section 6 can be regenerated rather than taken on trust.

### 8.5 Limitations

Several limitations qualify the results. (1) The programme-level evaluation used a single programme at one university; generalisation requires replication across programmes and institutions. (2) Completeness is measured at the level of skill mentions in vacancy text, which proxies presence rather than depth of competency mastery; it is nonetheless an objective, reproducible monitoring measure. (3) A learner cannot realistically complete all 246 recommended courses, so the practically meaningful output is the top 3 to top 5 list, not the full catalogue. (4) Output quality depends on the granularity of the source programme description: vague learning outcome text yields fewer extractable terms and biases recommendations towards broad coverage, whereas detailed, technology-explicit descriptions yield sharper, more targeted recommendations. (5) The HGT model overfits on long meta-paths and on the small CUA test set (30 nodes); added regularisation and a larger, more diverse graph would reduce this. (6) The labour-market snapshot is time-bound (Feb–Mar 2024) and IT-focused; periodic re-collection is needed to keep recommendations current.

Three further limitations concern the integration and evaluation methodology. First, the semantic integration depends on fixed similarity thresholds (τ₁ = 0.87 for competency–skill and τ₂ = 0.81 for skill–skill links); these were tuned to favour precision, but a single global threshold cannot be optimal for every skill family. Adaptive or per-cluster thresholds represent a natural refinement. Second, the evaluation reports point estimates of the ranking metrics on a single train/test partition. Because the competency test set is small, these estimates carry non-negligible variance. Although the consistency between the held-out metrics and the independent deployed-model application metrics (Section 6.7) is reassuring, the study does not include formal cross-validated significance testing, which would strengthen the comparative claims. Third, the framework is evaluated in a static, content-based setting: it does not model how recommendations would perform once learners act on them, how competency demand drifts over time, or how the graph should be updated incrementally as new vacancies and courses arise. A longitudinal, online evaluation with periodic graph refresh would be required to establish that the front-loaded competency gains observed here persist under realistic deployment, and this is the most important direction for validating the practical claims of this work. Addressing the second limitation mentioned above, this revision now reports bootstrap confidence intervals for the ranking metrics (Tables 7, 11), a paired bootstrap test for the HGT–HAN comparison, and a 5-fold cross-validation over the competency nodes, so the comparative claims rest on quantified uncertainty rather than single-partition point estimates.

## 9 Conclusion

This article presents a multilingual, multi-domain heterogeneous knowledge graph framework that integrates university curricula, labour market vacancies, and MOOCs, and recommends courses by predicting a continuous relevance weight on a competency-to-course relation. A property-graph ontology was formalised and instantiated for the three domains; skills were aligned across English, Russian, and Kazakh with a fine-tuned multilingual Sentence Transformer (best F1 among the compared models; improved Russian and Kazakh correlations after fine-tuning); skill communities were formed with agglomerative clustering (silhouette 0.271 → 0.285; erroneous clusters 19.7% → 14.0% after fine-tuning); and recommendation was solved as edge-weight regression with GraphSAGE, HAN, and HGT encoders, the HGT encoder being the strongest ranker (test NDCG@10 = 0.714; 0.987 with short meta-paths). Deployed in a Flask/Neo4j prototype and tested on a real programme, the system expanded the market-aligned competency profile by +36.2% with five courses and +170% in total, with the highest-value courses appearing first.

The contributions answer the five research questions: a coherent tri-domain multilingual ontology (RQ1); a fine-tuned multilingual skill-integration method with released evaluation data (RQ2); a clustering-based densification validated by a controlled comparison (RQ3); the first edge-weight-regression formulation on a cross-domain educational KG with an HGT-versus-baselines comparison and a meta-path ablation (RQ4); and a measurable, front-loaded improvement in curriculum completeness (RQ5). Future work will replicate the evaluation across programmes and universities, incorporate competency-depth signals, add regularisation and graph diversity to curb overfitting, refresh the labour-market snapshot, and extend the approach to corporate reskilling and upskilling.

## Data Availability

The original contributions presented in the study are included in the article/supplementary material, further inquiries can be directed to the corresponding authors.
